# Hereditary Neuromuscular Disorders in Reproductive Medicine

**DOI:** 10.3390/genes15111409

**Published:** 2024-10-30

**Authors:** Agnese Luglio, Elena Maggi, Francesco Nicola Riviello, Alessandro Conforti, Ugo Sorrentino, Daniela Zuccarello

**Affiliations:** 1 Medical Genetics Unit, IRCCS Azienda Ospedaliero-Universitaria di Bologna, 40138 Bologna, Italy; agnese.luglio@studio.unibo.it; 2 Clinica Eugin Modena, 41126 Modena, Italy; 3 U.O.C. Laboratorio di Genetica Medica, PO Di Venere-ASL Bari, 70012 Bari, Italy; 4 Department of Neuroscience, Reproductive Science and Odontostomatology, University of Naples Federico II, 80131 Naples, Italy; 5 Department of Women’s and Children’s Health, University Hospital of Padova, Via Giustiniani 3, 35128 Padova, Italy; 6 Unit of Medical Genetics and Genomics, San Bortolo Hospital, ULSS n.8 “Berica”, 36100 Vicenza, Italy; daniela.zuccarello@unipd.it

**Keywords:** neuromuscular disorders, reproductive medicine, preimplantation genetic testing, prenatal diagnosis, fertility, pregnancy, CMT, dystrophy, SMA, ALS

## Abstract

Neuromuscular disorders (NMDs) encompass a broad range of hereditary and acquired conditions that affect motor units, significantly impacting patients’ quality of life and reproductive health. This narrative review aims to explore in detail the reproductive challenges associated with major hereditary NMDs, including Charcot–Marie–Tooth disease (CMT), dystrophinopathies, Myotonic Dystrophy (DM), Facioscapulohumeral Muscular Dystrophy (FSHD), Spinal Muscular Atrophy (SMA), Limb–Girdle Muscular Dystrophy (LGMD), and Amyotrophic Lateral Sclerosis (ALS). Specifically, it discusses the stages of diagnosis and genetic testing, recurrence risk estimation, options for preimplantation genetic testing (PGT) and prenatal diagnosis (PND), the reciprocal influence between pregnancy and disease, potential obstetric complications, and risks to the newborn.

## 1. Introduction

Neuromuscular diseases (NMDs) comprise a broad and heterogeneous group of hereditary and acquired disorders that affect motor units (motor neurons, peripheral nerves, neuromuscular junctions, or muscle fibers). Although individually rare, these conditions are relatively common when considered collectively, with a prevalence of 220 per 100,000 and an incidence of 14.2 per 100,000 per year estimated in the UK [1]. The most common NMDs, listed by frequency, include Charcot–Marie–Tooth disease (CMT), Duchenne and Becker Muscular Dystrophies (*DMD* and BMD), Myotonic Dystrophy (DM1 and DM2), Facioscapulohumeral Muscular Dystrophy (FSHD1 and FSHD2), Spinal Muscular Atrophy (SMA), and Limb–Girdle Muscular Dystrophy (LGMD).

NMDs significantly impact patients’ quality of life, not only due to motor disability and challenges in daily living but also in the context of reproduction and family planning, as individuals with NMDs face additional challenges related to their parental aspirations. Therefore, it is crucial to provide this category of patients with comprehensive information on recurrence risk, reproductive options (such as preimplantation genetic testing/PGT and prenatal diagnosis/PND), the impact of pregnancy on the course of the disease, possible obstetric complications, and risks to the newborn [2]. Currently, this information is limited and makes genetic counseling and reproductive healthcare particularly complex and challenging [3,4]. The clinical management of NMDs is further complicated by the fact that they can manifest or be diagnosed during reproductive age, sometimes even during pregnancy.

The complexity of NMDs management, particularly in the reproductive context, requires a deep understanding of the genetic causes of the disease and the clinical and social implications for pregnancy and parenthood [2,3]. Molecular diagnosis (or confirmation) of NMDs is especially critical, not only to determine the recurrence risk and provide appropriate preconception counseling but also to enable proper pregnancy management and inform about available reproductive options and potential complications. Preimplantation genetic testing and prenatal diagnosis offer new possibilities for couples carrying known genetic variants, but it is important to inform patients that these options may not always be available [3,4]. Additionally, women with NMD have a higher risk of obstetric complications compared to the general population, and some patients report worsening or onset of symptoms that may not resolve after delivery [2,5,6,7].

The aim of this narrative review is to provide physicians and patients with the fundamental information they may need to understand and manage the nuances and reproductive challenges of NMDs. The concepts discussed here are summarized in Table 1.

## 2. Charcot–Marie–Tooth Disease (CMT)

### 2.1. Epidemiology and Clinical Features

Charcot–Marie–Tooth (CMT) disease refers to a heterogeneous group of hereditary disorders that affect peripheral nerves, leading to progressive muscle weakness and atrophy, primarily in the distal limbs. The estimated prevalence of CMT is approximately 1 in 2500 people, making it the most common inherited neuromuscular disorder [8,9].

CMT typically presents slowly progressive symmetric distal muscle weakness and atrophy, with onset generally in the first to third decade of life. Weakness and atrophy are most noticeable in the feet and hands, often leading to difficulties with walking and hand functions. Sensory loss, although often mild, can affect the senses of vibration, pain, temperature, and joint position [10,11]. Pain is not a common feature, but it can occur in some individuals [12]. The progression of CMT is generally slow but can vary significantly among individuals and different genetic subtypes. Over time, patients may develop more pronounced muscle atrophy and weakness, which can then spread to the lower legs and forearms. Balance problems and foot deformities, such as hammer toes, may become more prominent with age, necessitating orthopedic interventions or assistive devices [10,11]. Approximately 20% of patients with CMT eventually become nonambulatory, relying on wheelchairs for mobility [13]. Respiratory muscle weakness, particularly involving the diaphragm, can be observed in some CMT patients, with up to 37.7% experiencing symptoms such as obstructive sleep apnea [14]. Severe cases can lead to diaphragmatic dysfunction and respiratory failure, although this is rare [15]. Furthermore, fatigue and balance problems are prevalent in patients with CMT, with a substantial proportion reporting significant difficulty in daily activities [16]. These symptoms can have a considerable impact on daily activities, including pregnancy and the subsequent care of a newborn.

### 2.2. Genetic Diagnosis and Reproductive Risk

CMT is characterized by locus heterogeneity, with more than 80 genes identified as causative to date [17]. In order of frequency, the most common forms are CMT1A (*PMP22* duplication, accounting for more than 50% of genetic diagnosis), CMT1X (*GJB1* variants; X-linked, 10.7% of cases), CMT2A (*MFN2* variants, autosomal dominant, 7%), and CMT1B (*MPZ* variants; autosomal dominant, 6.7%) [18,19]. The subtype with the highest prevalence, CMT1A, results from the duplication of the *PMP22* gene on chromosome 17p11.2 (gene dosage effect) [20,21,22,23]. In the other genetic subtypes (particularly, *GJB1*, *MPZ*, and *MFN2*-related), there is no toxic gain-of-function but rather a loss-of-function mechanism [24,25,26,27,28].

Since CMT1A is the most common form, the initial step in the genetic diagnosis involves testing for duplication of the *PMP22* gene. *PMP22* duplication can be identified through targeted methods such as Multiplex Ligation-Dependent Probe Amplification (MLPA) or quantitative Polymerase Chain Reaction (PCR) [11], but also genome-wide approaches such as Chromosomal Microarray Analysis (CMA). If a *PMP22* duplication is not detected, the next step involves multigene panel testing [29,30]. If multigene panel testing does not yield a diagnosis, comprehensive genomic testing, such as Whole-Exome Sequencing (WES) or Whole-Genome Sequencing (WGS), should be considered [31].

CMT is primarily transmitted in an autosomal dominant manner, as mentioned above; individuals with these subtypes have a 50% chance of transmitting the disorder to their offspring. It is worth noting that clinical presentation can vary widely, even among family members with the same variant, due to incomplete penetrance and variable expressivity [25,27], complicating reproductive counseling and decision-making for patients. CMT1X, the X-linked form related to *GJB1* variants, is also relatively common [18]. A female carrier has a 50% chance of passing on the variant to her children, regardless of their gender. Hemizygous males will pass it on to all their daughters but none of their sons. Similarly to other X-linked conditions, hemizygous males typically exhibit more severe symptoms than heterozygous females. Females with CMT1X show a wide range of clinical manifestations, often milder than those observed in males. Due to this variability, genetic testing for *GJB1* in females—including prenatal and preimplantation settings—can identify carriers but cannot accurately predict the severity of the disease. Therefore, genetic counseling is crucial to help families understand the potential range of clinical outcomes and reproductive implications [24,32,33].

### 2.3. Prenatal Diagnosis and Preimplantation Genetic Testing

PND and PGT are widely applied in clinical practice for CMT, which is among the top 10 indications for PGT for monogenic defect (PGT-M) according to the 2018 data collection of the European Society of Human Reproduction and Embryology (ESHRE) [34]. For prenatal diagnosis, the same methodologies applied in postnatal settings can be used. Identification of a familiar variant and molecular confirmation in the affected or at-risk partner are generally required prior to performing PND. Specifically, for cases involving duplication of *PMP22*, methods such as MLPA, Comparative Genomic Hybridization array, and Single-Nucleotide Polymorphism array are routinely used in prenatal settings. This is different in the PGT context, as the methods used to detect *PMP22* duplications in the postnatal setting are unsuitable for embryo testing due to the insufficient amount of DNA from trophectoderm biopsy (5–10 cells)—or even polar body biopsy or single blastomere biopsy (single cell). Currently, the methodologies used for CMT embryo testing primarily rely, as for other monogenic disorders, on indirect analysis (haplotyping) rather than directly detecting Copy Number Variations (CNVs) of *PMP22*. Techniques such as fluorescent PCR amplification of Short Tandem Repeat (STR) polymorphic markers and karyomapping combined with genome-wide linkage analyses are utilized to determine the at-risk haplotype (associated with *PMP22* duplication). This indirect approach allows the identification of embryos that do not carry the disease-associated haplotype [35,36,37]. Linkage analysis, which relies on the presence of positive family members to construct at-risk haplotypes, is not feasible for de novo CMT1A patients. Additionally, chromosomal exchanges near or within the mutated region can complicate the diagnosis, potentially leading to the identification of unaffected embryos. The linkage analysis process for *PMP22* duplication is further complicated by issues such as triple copy number inference, low resolution, and a significant risk of misdiagnosis [37].

### 2.4. Fertility and Pregnancy

Recent studies on pregnancy outcomes in CMT present a generally positive outlook. In particular, Rudnik-Schöneborn et al. (2020) reported favorable pregnancy outcomes in their cohort study, indicating no significant increase in obstetric and neonatal complications compared to the general population [38]. However, other studies have identified certain risks. For example, Pisciotta et al. (2020) highlighted an increased incidence of placental abnormalities (an increased rate of placenta previa, occurring in 1.6% of cases compared to 0.4% in the reference population) [39]. Furthermore, Skorupinska et al. (2023) found a higher rate of urinary tract infections, with 4.1% of CMT pregnancies affected compared to 0.8% in the general population [40]. Abnormal fetal presentations are also a concern. Hoff et al. (2005) and Pisciotta et al. both documented a higher occurrence of abnormal fetal presentations in CMT pregnancies [38,41]. In Hoff et al., presentation anomalies were twice as common in CMT pregnancies compared to controls (9.3% vs. 4.5%), while Pisciotta et al. found that 8.4% of CMT pregnancies involved non-vertex presentations, significantly higher than 4.5% in the general population [38,41]. Preterm births are also a significant risk. Pisciotta et al. reported that preterm delivery occurred in 20.3% of CMT pregnancies, compared to 6.9% in the general population [39]. Furthermore, Hoff et al. (2005) noted an increased need for operative deliveries, including cesarean sections and the use of forceps, in CMT pregnancies (the overall rate of operative delivery, including cesarean section or the use of forceps or vacuums was 29.6% in CMT pregnancies versus 15.3% in controls) [41]. Postpartum hemorrhage was also more common (12.0% in CMT vs. 5.8%). In approximately one-third of pregnancies, patients experience a worsening of their symptoms that generally do not remit after delivery. Symptoms such as weakness, sensory loss, cramps, pain, fatigue, and balance problems are commonly reported to deteriorate [6,38,40]. For example, Rudnik-Schöneborn et al. found that 37.8% of pregnancies in their study reported a worsening of CMT symptoms [38]. Skorupinska et al. (2023) observed that 36.8% of pregnancies reported worsening symptoms, particularly in walking (71.4%) and balance (58.7%) [40]. A different trend was observed in the study by Pisciotta et al., where only 16.3% of the patients reported worsening symptoms during pregnancy (9.3% of pregnancies) [39]. Interestingly, this study also documented an improvement in 50% of cases without occurrence in subsequent pregnancies. The lower rate of reported symptom deterioration could be attributed to differences in the study population or the design of the study, including the method of data collection (i.e., online multicenter self-reported questionnaire vs. in-person data collection). Despite the potential for symptoms worsening, most CMT patients would consider pregnancy again and express a positive attitude. Many emphasize the importance of adequate support and careful management during and after pregnancy to mitigate the challenges associated with the disease [38,42]. Finally, while some studies indicate an increased risk of specific complications such as placental abnormalities, urinary tract infections, abnormal fetal presentations, preterm birth, and operative deliveries in CMT pregnancies, the overall outcomes remain favorable. However, it is complex to compare the results across different studies due to variations in study populations and designs. More prospective studies are needed to comprehensively clarify these risks. Nonetheless, the general sentiment among CMT patients is positive, with many willing to face pregnancy again and offering advice to others about how to manage potential challenges effectively.

## 3. Dystrophinopathies (*DMD*, BMD, and *DMD*-Associated DCM)

### 3.1. Epidemiology and Clinical Features

Dystrophinopathies are a group of allelic disorders caused by pathogenic variants in the *DMD* gene on chromosome X. They are the most common hereditary muscular dystrophies, with an overall incidence of 1 in 5000 to 6000 live male births [42]. The clinical spectrum includes Duchenne muscular dystrophy (*DMD*), Becker muscular dystrophy (BMD), and *DMD*-associated dilated cardiomyopathy (DCM) [42]. Approximately two-thirds of patients inherit the causative variant from their mother, while de novo variants account for about a third of cases [43]. Currently, there is no precise data on carrier frequency. However, assuming a population in Hardy-Weinberg equilibrium and an incidence of 1:5000 for DMD/BMD, the carrier frequency can be estimated at approximately 1:2500 [44]. Notably, a recent population screening study revealed a higher carrier frequency of 1:1374 [44]. *DMD* in women is very rare (<1 per million) and is generally limited to clinical cases involving individuals with Turner syndrome [45].

*DMD* manifests itself in early childhood with an onset between 2 and 5 years of age, characterized by delayed motor milestones, waddling gait, difficulties in postural transitions, and frequent falls. The disorder mainly affects the proximal muscles, causing difficulty climbing stairs, jumping, and running [46]. The disease progresses rapidly, with loss of independent ambulation occurring between 6 and 13 years, followed by the rapid development of joint contractures and scoliosis. Cardiomyopathy and respiratory failure are the leading causes of death in young adults. Nowadays, life expectancy is extending into one’s late twenties and early thirties [47,48]. Patients with *DMD* exhibit a significant increase in serum creatine phosphokinase (CPK) levels, typically exceeding 10 times the normal reference values [47,49]. Histological examination of muscle tissue reveals non-specific dystrophic changes, which eventually lead to infiltration of connective and fat tissue. Immunohistochemical staining for dystrophin and immunoblotting (Western blot) on muscle biopsy shows a complete or near-complete absence of dystrophin (less than 5% of the normal protein quantity) [50].

BMD presents with a milder phenotype and later onset, with patients typically maintaining ambulation into late adolescence or adulthood. Despite milder skeletal muscle involvement, DCM is a frequent cause of morbidity and is often the most common cause of death in patients with BMD, manifesting later in life [51,52]. Muscle biopsy shows a greater representation of dystrophin compared to *DMD*, while CPK levels show a more moderate increase.

Some forms present exclusively with isolated DCM, without the typical skeletal muscle manifestations observed in DMD or BMD [53].

Except for specific cases (e.g., X chromosome monosomy, X-autosome translocation, other rearrangements involving Xp21.2, or biallelic variants), females typically do not exhibit classic muscular involvement [54,55]. The clinical variability in females is primarily explained by different patterns of the inactivation of the X chromosome. Female carriers, including asymptomatic cases, can show elevated serum CPK (in about 30–50% of cases) and mild reductions in the amount of dystrophin in muscle tissue (generally > 60%) [49,50]. Since muscle symptoms are relatively rare in female carriers (2.5–10%) [56], the primary concern is the potential development of cardiomyopathy. Cardiac involvement is often subclinical and ranges from 45% to 70% of female carriers, depending on the assessment method used [57,58,59]. Given this variability, regular cardiac monitoring is recommended, even for those without symptoms, to detect and manage cardiomyopathy early [57,58,59].

### 3.2. Genetic Diagnosis and Reproductive Risk

The *DMD* gene, located on the X chromosome in the Xp21.2 region, encodes dystrophin, a key muscle protein [60]. The most common molecular defect in the *DMD* gene is the deletion of one or more exons, occurring in 60–70% of *DMD* and BMD cases. Duplications account for 5–10%. Finally, Single-Nucleotide Variants (SNVs) account for approximately 25% of causative variants [61,62]. Rarely, dystrophinopathies are caused by more complex rearrangements or deep intronic changes (<2%) [63]. The “reading frame rule” explains the difference between the more severe *DMD* and the milder BMD phenotype. According to this rule, if a variant disrupts the reading frame (out-of-frame variant), it results in a nonfunctional, truncated dystrophin protein, leading to *DMD* (complete or near-complete absence of functional protein). Conversely, variants that maintain the reading frame (in-frame variants) allow the production of a shorter but partially functional dystrophin, leading to the phenotypically milder BMD [43].

The diagnostic approach should involve a gene-targeted deletion/duplication analysis, such as MLPA [64,65]. Other techniques, such as CMA, can also detect CNVs involving *DMD*, typically identifying larger deletions or duplications compared to intragenic deletions/duplications [64]. If the MLPA test is negative, further analyses are necessary to investigate the remaining types of variants, such as point variants or small insertions/deletions [64,66]. For healthy women without a family history of dystrophinopathy, carrier screening is recommended according to the American College of Human Genetics and Genomics (ACMG) [67]. The carrier screening test is typically performed using MLPA analysis.

Dystrophinopathies are inherited in an X-linked manner, where female carriers have a 50% chance of passing the causative variant to their offspring. Hemizygous males will pass it on to all their daughters but none of their sons. As for other X-linked disorders, heterozygous females may present with variable phenotypes. More often, females remain asymptomatic. Genetic testing cannot predict the potential development of signs or symptoms of the disease in women [64]. Therefore, genetic counseling is essential to discuss these potential risks with families.

### 3.3. Current Treatment and Emerging Therapies

In the context of reproductive medicine, genetic counseling should not only address genetic risks but also include discussions of current and emerging therapeutic options. Although there is currently no cure for DMD, several therapeutic strategies are available. Traditional treatment focuses primarily on treating symptoms rather than addressing the underlying pathogenic mechanism [68]. The current gold standard is corticosteroid therapy, which has been shown to improve both quality of life and survival [68]. Symptomatic treatment also includes physical therapy, management of cardiac and respiratory failure, orthopedic complications (such as scoliosis and corticosteroid-induced osteoporosis), and maintaining dietary balance to support overall health [68]. However, therapies that directly target the causal genetic defect are of critical importance [68,69]. A significant advancement in *DMD* treatment has been the development of exon skipping therapies. These therapies use antisense oligonucleotides to skip specific exons in the *DMD* gene, restoring the reading frame and allowing for the production of a truncated yet functional protein. Several exon-skipping therapies have received regulatory approval [68,69]. Despite producing a small amount of shorter protein, these therapies have been shown to slow disease progression, preserve muscle function, and improve both quality of life and life expectancy [68,69]. However, exon skipping is variant-specific and cannot be applied to all patients [69]. Emerging approaches, such as gene therapy using adeno-associated virus (AAV) vectors, are currently being tested in clinical trials [68,69]. These therapies aim to deliver micro-dystrophin genes, which encode a smaller but functional protein, offering the potential for systemic treatment in all muscle tissues [68,69]. CRISPR-Cas9 genome editing is also under investigation, with the goal of permanently correcting the genetic variants responsible for *DMD* [68,69]. Incorporating this information into genetic counseling is essential for families making informed reproductive decisions, as these treatments can significantly influence the long-term outlook of people affected by DMD.

### 3.4. Prenatal Diagnosis and Preimplantation Genetic Testing

A confirmed molecular diagnosis is crucial for female carriers to be able to access PGT or PND. Preferentially, PND is performed via chorionic villus sampling (CVS) rather than amniocentesis (AC), as it allows earlier results [64]. The same genetic tests used in postnatal settings are applied to DNA obtained using CVS or AC in the prenatal setting [64]. According to current guidelines, PND should be limited to male fetuses [64]. However, PND can be considered in female fetuses on a case-by-case basis, with options thoroughly discussed with the couple [64].

Historically, sex-based selection was the initial method used in PGT for *DMD*, aimed at reducing the risk of affected offspring by transferring only female embryos [70]. However, this approach had limitations, as it excluded potentially healthy male embryos and reduced the embryo pool for transfer by half (only females) [70,71]. Currently, the standard approach in PGT-M primarily involves indirect linkage analysis to identify the at-risk haplotype, as methods such as MLPA are less feasible due to the small amount of DNA obtained from embryo biopsies. However, indirect analysis may lead to misdiagnosis in cases of recombination events, or haplotype reconstruction may not be possible if no family members are available. To improve accuracy, direct mutation analysis using PCR-based techniques or NGS is often performed in parallel. NGS offers additional advantages, as it can simultaneously assess polymorphic markers and identify pathogenic variants, including CNVs, depending on the platform used. This dual capability improves diagnostic accuracy, making NGS an increasingly effective tool [70].

### 3.5. Fertility and Pregnancy

According to the available data, fertility does not appear to be affected in individuals with dystrophinopathies by the natural course of the disease. The available data on male fertility in *DMD*/BMD are limited, largely due to early mortality and the lack of focus on reproductive health in these patients [72]. Case reports indicate that fertility may not be inherently impaired [73,74]. However, Eggers et al. (1995) reported a significantly lower reproductive fitness in BMD patients compared to LGMD males, potentially due to social and economic factors as well as biological differences, although the latter remains speculative [75]. The introduction of long-term corticosteroid therapy, the current gold standard of care, has increased survival and allowed more patients to reach reproductive age; however, this treatment is associated with several side effects, including delayed puberty and hypogonadism, resulting in iatrogenic infertility [74,76]. Testosterone therapy in *DMD* patients was recently introduced in the Duchenne Muscular Dystrophy Care Considerations (2018) and has shown promising results in improving both quality of life and biochemical parameters related to spermatogenesis, offering encouraging prospects for future fertility [74,76]. Further studies, including sperm analyses, are needed to confirm these effects.

The evidence on potential pregnancy-related risks for female carriers is limited too. In most cases, pregnancy and postpartum proceed without complications. Since many heterozygous women do not exhibit obvious signs or symptoms of the disease, it is likely that the course of pregnancy and postpartum is comparable to that of the general population. Furthermore, experimental studies suggest that estrogen, progesterone, and corticosteroids may have a protective effect on muscle during pregnancy [77]. Some authors, based on their professional experience, report that about two-thirds of symptomatic carriers experience a worsening of symptoms, possibly due to increased weight and cardiovascular and metabolic demands; this worsening could persist into the postpartum period, possibly due to hormonal changes [7]. Furthermore, some case reports document severe complications in pregnancy. In 2001, a case was reported of a 25-year-old female carrier who developed severe cardiac failure during the third trimester of pregnancy, requiring mechanical circulatory support and transplantation [78]. Additionally, four other cases of peripartum cardiomyopathy (PPCM) have been described [55,79,80]. Ware et al. (2016) showed in their study that PPCM and dilated cardiomyopathy share the same genetic basis and that *DMD* variants may be a rare cause of PPCM (1 in 172 women analyzed in their study) [81]. Although the specific evidence for *DMD* carriers is limited, careful follow-up for these patients should be planned to manage potential complications such as worsening muscle weakness and exacerbating cardiomyopathy [82].

## 4. Myotonic Dystrophy (DM)

### 4.1. Epidemiology and Clinical Features

Myotonic dystrophy type 1 (DM1) and type 2 (DM2) are the most common muscular dystrophies in adulthood and represent the second most frequent dystrophy after *DMD*/BMD. The prevalence of DM1 exhibits considerable variation, ranging from 5 to 20 cases per 100,000 individuals worldwide [83]. According to a recent meta-analysis, the overall pooled prevalence of DM is approximately 10 per 100,000. When analyzing DM1 and DM2 separately, the estimated pooled prevalence is 9.27 and 2.29 per 100,000, respectively [83].

DM1 affects smooth and skeletal muscles, as well as the eye, heart, endocrine system, and central nervous system. The clinical outcomes, which range from mild to severe, have been classified into three partially overlapping phenotypes: mild, classic, and congenital [84]. Mild DM is characterized by cataracts and mild myotonia (sustained muscle contraction); life expectancy is normal. Classic DM1 involves muscle weakness and atrophy, myotonia, cataracts, and often cardiac conduction abnormalities; affected adults may experience physical disability and a reduced lifespan. Congenital DM1 manifests itself with hypotonia and severe generalized weakness at birth, often leading to respiratory failure and early death; intellectual disability is also common [85]. DM2 typically includes symptoms of myotonia and muscle dysfunction, such as proximal and axial weakness, myalgia, and stiffness. Less frequently, it can involve posterior subcapsular cataracts, cardiac conduction defects, insulin-resistant type 2 diabetes mellitus, and other endocrine abnormalities. The onset typically occurs in the third to fourth decade [86].

The penetrance of both conditions, DM1 and DM2, is age-dependent, reaching nearly 100% by the age of 50. As with other repeat expansion disorders, DM1 can exhibit anticipation—unlike DM2—where the age of onset becomes earlier and the severity of the condition increases in successive generations. In extreme cases of anticipation, congenital forms of DM1 may occur [87,88].

### 4.2. Genetic Diagnosis and Reproductive Risk

Both DM1 and DM2 involve repeat expansions and a consequent toxic RNA gain-of-function mechanism. DM1 is caused by the expansion of a (CTG)n trinucleotide repeat in the 3′ untranslated region of the *DMPK* gene (19q13.32). The severity of the disease and the age of onset correlate with the number of CTG repeats, which can range from 50 to over 3000 [89]. This molecular correlation explains the phenomenon of anticipation observed in DM1, which occurs remarkably more frequently on the maternal allele [87,88].

On the other hand, DM2 is caused by an expansion of a (CCTG)n tetranucleotide repeat in intron 1 of the *CNBP* gene (3q21.3). Unlike DM1, DM2 does not exhibit the phenomenon of anticipation, as the repeat expansion in DM2 does not typically increase in size in successive generations and does not correlate with disease severity [90].

The genetic testing methodologies for DM1 and DM2 are similar, and both are based on PCR-based techniques and Southern blot analyses. For DM1, the first step generally involves PCR and fragment length analysis that allows the detection of normal and smaller expanded alleles (up to 100–150 CTG). If only one allele size is detected by conventional PCR, a second step with triplet-repeat Primed (TP)-PCR and Southern blotting is necessary to confirm the presence of expansion and accurately determine the size of larger expansions [91]. Normal *DMPK* alleles, which are stable and non-pathogenic, have up to 34 CTG repeats; pre-mutation alleles, which are associated with increased risk of expansion across generations, span from 35 to 49 repeats; full-mutation alleles contain more than 50 repeats [92]. For DM2, testing is more challenging due to several factors. The complex-repeat tract at the *CNBP* locus contains flanking (TG)n, (TCTG)n, and (CCTG)n repeats, where only the CCTG expansion is associated with the disease [91]. In addition, the size of pathogenic alleles is characterized by a wider range (from 75 to more than 11,000). Therefore, a combination of analytic methods is usually applied [91]. Similarly to DM1, a first step with PCR and fragment-length analysis allows the detection of lower-range size repeats. Southern blotting is then used to detect expansions and estimate their size. Finally, a repeat-primed PCR assay is applied to confirm the presence of expansion. For DM2, normal non-pathogenic alleles contain up to 26 CCTG repeat units, and pathogenic alleles typically have 75 or more, sometimes exceeding 11,000 (mean 5000) [91]. It is important to specify the clinical indication and the likelihood of DM diagnosis when requesting genetic analysis. In fact, this information guides the interpretation and selection of the most appropriate testing strategy. An accurate clinical context improves diagnostic accuracy and aids in providing appropriate genetic counseling [91].

### 4.3. Prenatal Diagnosis and Preimplantation Genetic Testing

PND for Myotonic Dystrophy, performed on DNA extracted from CVS or amniotic fluid, follows the same analytical steps as postnatal testing. Maternal DNA is required to rule out maternal contamination. Additionally, a sample from the unaffected parent may be requested to verify the PCR results, particularly when the fetus shows two alleles within the normal size range [91].

In the 2018 ESHRE PGT Consortium data collection, DM1 is one of the main indications for PGT-M [34]. Standard repeat or flanking PCR to detect the normal *DMPK* allele of the affected parent is commonly employed in PGT-M for DM1 due to reliability issues in detecting the expanded allele [93]. This method has been useful only for couples with informative normal alleles, where the normal allele size of the affected individual differs from that of the unaffected partner. Even when a couple’s normal alleles are informative, if the normal allele of the affected parent is not observed in an embryo and there are not enough informative markers to produce unequivocal haplotypes, the embryo cannot be transferred because it is not possible to determine whether the embryo is affected or unaffected. A study from 2019 presented an alternative strategy, which uses whole genome amplification followed by TP-PCR detection of expanded *DMPK* alleles in parallel with single-tube haplotype analysis of 12 closely linked and highly polymorphic microsatellite markers [94]. Bidirectional *DMPK* TP-PCR reliably detects repeat expansions even in the presence of non-CTG interruptions at both ends of the expanded allele. Misdiagnoses, diagnostic ambiguity, and the need for couple-specific test customization are further minimized by using multi-marker haplotyping, preventing the loss of potentially unaffected embryos for transfer [94].

### 4.4. Fertility and Pregnancy

In DM1, infertility affects both sexes due to various endocrine and gonadal dysfunctions, although the underlying pathogenic mechanisms are only partially understood [95]. In men with DM1, hypogonadism is common, with abnormalities in the hypothalamic–pituitary–testicular axis, leading to reduced testosterone levels, elevated gonadotropins (FSH, LH), and impaired spermatogenesis. Testicular atrophy occurs in 65.5% of patients, significantly contributing to fertility issues [96]. Erectile dysfunction is present in 72% of male DM1 patients, with 64% showing compensated hypogonadism (elevated LH and normal testosterone levels) and 8% suffering from primary hypogonadism (increased LH and decreased testosterone) [97]. Furthermore, reduced sperm function is commonly observed. In a recent study by Ergoli et al., 50% of evaluated DM1 men show hypoposia and azoospermia, while the remaining 50% present with oligo-asteno-teratozoospermia [95].

Women also experience reduced fertility due to menstrual disturbances, a diminished ovarian reserve, and a poor response to ovarian stimulation [98]. In DM1, there is an increased risk of obstetric complications, attributed to the involvement of both skeletal and visceral smooth muscles (including uterine atony and dysfunction of the placental, uterine, tubal, and bladder smooth muscles) [6,99,100,101]. These complications include an increased rate of ectopic pregnancy (about 4%) and placental anomalies (9–11%), particularly placenta previa, which can carry a significant risk of massive hemorrhage during delivery [6,99,101]. Furthermore, patients experience a higher incidence of urinary tract infections (9–19%) [6,101]. Polyhydramnios (17–25%) is another common complication, typically identified in the seventh month of gestation and seen exclusively in fetuses affected by the congenital form of DM1, as a consequence of severely reduced fetal movements [6,101]. Furthermore, there is an increased risk of preterm labor (31–50%), instrumental delivery (15%), cesarean section (37%), abnormal fetal presentation (35%), and peripartum hemorrhage (17%) [6,99]. In 21–36% of pregnancies in women with DM1, the newborn is affected by the congenital form of DM1, which often results in significant neonatal complications and a high rate of admission to neonatal intensive care units (90%) [6,99]. Perinatal loss, defined as in utero death after 28 weeks of gestation or postnatal death within the first month, occurs in up to 15% of cases [101]. Regarding disease progression, some studies report a worsening of symptoms during pregnancy and six months after delivery, particularly with respect to myotonia, pain, mobility, and limitations of activity, with some women not experiencing subsequent remission [6,100]. However, other studies do not report significant changes in symptoms during or after pregnancy [6].

Although DM2 generally follows a more benign course than DM1, some features, including male hypogonadism and fertility issues, are comparable. Male fertility in DM2 is significantly affected, with primary testicular failure being common. Approximately 65% of men with DM2 exhibit elevated FSH levels, along with low or low-normal testosterone and oligospermia [102]. Despite these findings, there is limited evidence to suggest any direct impact on fertility in females. The course of pregnancy in patients with DM2 is generally less complicated than in DM1; however, there is an increased risk of obstetric complications, including preterm labor (17–50%) and urinary tract infections (7.6%) [6,90]. In some cases, pregnancy may reveal the disease, with 14% of women experiencing the onset of symptoms during gestation [6]. The worsening of symptoms, particularly in the postpartum period, is also observed [100]. Unlike DM1, there are no documented cases of congenital DM2 [6,90]. The effect of pregnancy on cardiac diseases in DM is unknown. Respiratory complications are common in DM1 due to weakness and myotonia of the respiratory muscles, pharyngoesophageal weakness, and reduced respiratory capacity, which can lead to alveolar hypoventilation and respiratory failure. Regional anesthesia is preferred to general anesthesia [6,103].

## 5. Facioscapulohumeral Muscular Dystrophy (FSHD)

### 5.1. Epidemiology and Clinical Features

Facioscapulohumeral dystrophy (FSHD) is the third most frequent muscular dystrophy after *DMD* and DM, with an estimated prevalence of approximately 1 in 8000 to 1 in 22,000 [104,105,106].

FSHD is clinically characterized by progressive weakness and atrophy involving facial muscles, shoulders, and scapular girdle (the most affected region), pelvic girdle, lower abdominal muscles, and lower leg (pretibial muscle with footdrop) [107,108,109]. Asymmetry in muscle weakness is common [110]. The earliest signs often include sleeping with partially open eyes and difficulty whistling or sucking [108]. Classic facioscapulohumeral involvement occurs in approximately 70–85% of patients, leading to a characteristic appearance that includes full or slightly everted lips (facial weakness), winged scapula, straight clavicles, vertical or reversed anterior axillary fold secondary to scapular girdle involvement, protruding abdomen, and lumbar hyperlordosis due to abdominal weakness [108,109]. Some individuals may present with variant phenotypes that differ from the classic form, such as scapulohumeral dystrophy with facial sparing, severe infantile onset with rapid progression, association with cognitive impairment and epilepsy, and atypical forms due to the mosaic distribution of D4Z4 repeat lengths [111,112,113,114,115]. Extra muscular manifestations can be present, including retinal vasculopathy (vision not affected), exudative retinopathy that can lead to retinal detachment and vision loss, sensorineural hearing loss, and cardiac arrhythmias (rarely symptomatic) [116,117,118,119]. The onset of the disease usually occurs during adolescence or young adulthood, although this can vary widely. This is often a stage in life when people may already be considering family planning and parenting [114,115,120]. The disease usually presents a slow progression and a relatively ‘benign’ course, which does not affect life expectancy. However, approximately 20% of individuals eventually become wheelchair-dependent [121]. Respiratory involvement is generally not significant; however, 9–38% of patients may develop restrictive lung disease, and 1–3% may require non-invasive ventilatory support [122,123].

### 5.2. Genetic Diagnosis and Reproductive Risk

The molecular mechanism of FSHD is rather unique. The disease is caused by the inappropriate expression, in skeletal muscle, of *DUX4*, a transcription factor expressed in the embryo and normally repressed in adult somatic cells [124]. DUX4 exerts a toxic effect on muscles, increasing oxidative stress and apoptosis, interfering with myogenesis, and eventually causing muscle atrophy [125,126]. In more than 95% of cases (defined as FSHD1), the ectopic expression of *DUX4* is caused by the co-occurrence of a contraction of the D4Z4 macrosatellite repeats (each D4Z4 unit contains a copy of the *DUX4* gene) in region 4q35 in the range of 1–10 units (from 8–100 in the general population) and a genetic background of predisposition due to the presence of the 4qA permissive haplotype in cis. The D4Z4 repeat contraction decreases the DNA methylation of the locus, opening the chromatin structure and leading to the expression of the most distal copy of *DUX4*. The simultaneous presence of a 4qA permissive allele, distal to *DUX4* and containing its 3′ UTR, enables stabilization and translation of mRNA by acting as a functional polyadenylation site [125]. In contrast, FSHD2 patients, which are less than 5% of all FSHDs, show D4Z4 repeats within the borderline-normal range (8–20), and hypomethylation of the locus is caused by a pathogenic variant in a chromatin modifier gene located on a different chromosome (i.e., *SMCHD1* on 18p, *DNMT3B* on 20q, or *LRIF1* on 1p) [127,128,129,130,131]. In both forms, which are clinically indistinguishable, *DUX4* de-repression eventually occurs. It should be noted that in both forms a 4qA permissive allele is required to manifest the disease and that *SMCHD1*, *DNMT3B,* and *LRIF1* may act as modifiers of disease severity in FSHD1 [132]. The genetic basis of FSHD is further complicated by several factors: (i) the recombinogenic nature of the 4q35 region [133]; (ii) the presence of high homology between the subtelomeric regions of 4q and 10q, which allows for the possibility of translocations (these result from certain founder events during human evolution rather than recurrent events) [134]; and (iii) the potential presence of D4Z4 allele mosaicism due to postzygotic array contractions in the early stages of embryogenesis [135]. These factors can complicate the interpretation of genetic test results, particularly in preimplantation and prenatal settings [120].

The diagnostic algorithm for FSHD first requires a genetic analysis to determine the number of D4Z4 repeat units on chromosome 4q [136,137]. Southern blotting is the most commonly used method and allows for accurate sizing and chromosomal specificity of D4Z4 fragments. However, it is labor intensive, requires large amounts of DNA, and the analysis takes more than a week to complete [137]. However, analysis and interpretation present several challenges. Southern blot analysis is susceptible to false positive and false negative results. False negatives can be due to (i) the presence of proximal deletions to the D4Z4 repeats, which include the site for the p13E-11 probe, (ii) the presence of hybrid repeat arrays, or (iii) cases of FSHD2. False positives, on the other hand, are due to (i) the presence of shortened D4Z4 fragments on 10q (mistakenly identified as pathogenic) or (ii) shortened D4Z4 on non-permissive 4qB haplotypes [137]. It is essential to gather detailed clinical information at the time of DNA referral to mitigate false results. If Southern Blot or D4Z4 repeat analysis yields a negative result, subsequent tests should include methylation analysis and sequencing analysis focusing on the *SMCHD1*, *DNMT3B*, and *LRIF1* genes [137,138].

FSHD is primarily inherited in an autosomal dominant manner (FSHD1, >95% of cases). Therefore, the recurrence risk for most individuals is 50% for each conception, regardless of the sex of the offspring. On the other hand, FSHD2 exhibits digenic inheritance, as it requires the co-occurrence of a permissive allele in 4q and a pathogenic variant in a chromatin modifier gene affecting the D4Z4 region, located on different chromosomes. Therefore, the recurrence risk for the child of an individual with FSHD2 is 25% of being affected and 50% of being a heterozygous carrier, assuming that the other parent is healthy and not a carrier of either D4Z4 contractions or pathogenic variants in D4Z4-chromatin-modifier genes.

### 5.3. Prenatal Diagnosis and Preimplantation Genetic Testing

PND and PGT for FSHD are technically feasible but complex due to the underlying molecular mechanism of the disease [108,126]. PND for FSHD1 is feasible and used in clinical practice. The diagnosis is typically based on Southern blot analysis or LR-PCR to determine the size of the D4Z4 repeat array, as in the postnatal setting. Due to the time required for results (a few weeks), PND is performed preferentially in the first trimester (CVS) [120,138,139].

The Southern Blot method, typically used for FSHD testing, is not suitable for PGT due to the large amount of DNA required (>500 ng) [120,140]. Thus, PGT for FSHD1, as for other monogenic diseases, relies on indirect analysis based on haplotyping. Traditional methods are based on micro-satellite Short-Tandem Repeat marker (STR)-PCR. These methods are complicated by the telomeric position of the D4Z4 array on chromosome 4q, which prevents the inclusion of distal microsatellite markers. Consequently, traditional indirect tests for FSHD1 only use markers proximal to the D4Z4 locus, leading to an error rate exceeding 5% due to the high rate of recombination that went undetected [133,141]. Newer approaches, such as the OnePGT method, use NGS and SNPs as markers for haplotyping, reducing the risk of misdiagnosis (<5%). Despite these advances and the increased reliability of PGT for FSHD1, prenatal confirmation of PGT results is still recommended [120,133,141].

Conversely, PND and PGT are currently not available in clinical practice for FSHD2. In fact, although it is technically possible to identify a known pathogenic variant in genes associated with FSHD2 through both PND and PGT, it is not possible to predict the risk of the fetus developing the disease. This is due to the fact that it is not feasible to determine the number of D4Z4 repeats in the borderline range typical of FSHD2 (8–20) in these settings. Without the ability to identify the predisposition genotype, it is not possible to adequately establish the risk of developing the disease [120,138].

This topic has been extensively and brilliantly covered by Vincenten et al. in their 2022 review [120].

### 5.4. Fertility and Pregnancy

Fertility does not appear to be affected in FSHD. Studies suggest that women with FSHD can conceive and carry pregnancies to term with favorable outcomes, similarly to women in the general population, with no increased risk of ectopic pregnancy or miscarriage [120,142]. However, an increased incidence of certain obstetric complications has been observed. The cesarean and instrumental delivery rates are higher. The increased rate of cesarean deliveries reached statistical significance in the study by Ciafaloni et al., where the total rate of cesarean deliveries was 23.8% compared to 16.9% in the general population. Instrumental deliveries were also more common, with rates of 27% in patients with FSHD compared to 11.6% in the general population [120,142]. This increase in operative deliveries is attributed to the abdominal and pelvic muscle weakness characteristic of FSHD, which can complicate the second stage of labor [120]. In addition, there appears to be an increased risk of low birth weight (16.4% vs. 5.6%) [142], although this finding has not been confirmed by a more recent study by Awater et al. [6]. The same studies did not show an increased risk of congenital malformations or neonatal deaths specifically related to FSHD [6,142,143]. Regarding the course of the disease, 12–24% of pregnancies resulted in worsening of the symptoms, which generally did not resolve postpartum. Symptoms included increased general weakness, frequent falls, difficulty carrying the infant, and increased pain [120,142,143]. Due to the increased likelihood of operative deliveries, a comprehensive delivery plan should be established early in the pregnancy, including considerations about the type of anesthesia (regional anesthesia is preferred over general anesthesia) [120,142]. Although ventilatory and cardiac involvement is not common in FSHD, frequent monitoring of pulmonary function is recommended, particularly in patients with severe scoliosis or lumbar hyperlordosis. Forced vital capacity (FVC) measurements should be performed at least once per trimester and before delivery. Referral to specialists in pulmonary or sleep medicine is advised for patients with compromised pulmonary function (FVC <60% or a >15% reduction in supine compared to seated FVC). Routine cardiac monitoring is generally not required unless specific symptoms arise [120]. It should be noted that about 50% of women with FSHD did not know the diagnosis at the time of pregnancy [142,143]. Although studies reporting this percentage are dated and the application of genetic testing and counseling is now more widespread, it is important to emphasize that some patients of reproductive age may still be unaware of their diagnosis. Therefore, reproductive medicine professionals must be familiar with the key symptoms of the disease, allowing timely patient management and proper planning and care for pregnancy. Finally, despite the challenges and possible complications experienced, 90% of the women expressed that they would choose to become pregnant again [142].

## 6. Spinal Muscular Atrophy (SMA)

### 6.1. Epidemiology and Clinical Features

Spinal Muscular Atrophy (SMA) is characterized by progressive degeneration of motor neurons in the anterior horn of the spinal cord and the nuclei of the brain stem [144]. SMA has an estimated worldwide incidence of approximately 1 in 10,000 live births and a prevalence of approximately 1–2 per 100,000 individuals. The carrier frequency is generally estimated between 1 in 40 and 1 in 60. However, these epidemiological data show significant variability among different ethnic groups. Carrier frequency is estimated to be lower among individuals of sub-Saharan African (1 in 100) and Hispanic (1 in 70) descent and higher in European and Asian populations (about 1 in 45–50). In some genetic isolates or communities with high rates of consanguinity, even higher carrier frequencies have been observed [145].

SMA presents in various clinical forms, classified based on age of onset and severity of symptoms: SMA type 1 (infantile, also known as Werdnig-Hoffmann disease), SMA type 2 (intermediate), SMA type 3 (juvenile, Kugelberg-Welander disease), and SMA type 4 (adult) [146]. SMA Type I, the most severe form, onsets within the first six months of life. Infants with Type I experience severe hypotonia and difficulties with breathing and swallowing and never develop the ability to sit independently. Without medical intervention, the prognosis is poor, with a high mortality rate in the first two years. The onset of SMA Type II usually manifests between 6 and 18 months of age. Children with this condition can sit without support but are unable to walk unaided. They often develop complications such as scoliosis and respiratory problems. SMA Type III manifests itself after 18 months and varies in presentation from moderate to severe. Patients with Type III are generally able to walk initially, but many lose this ability over time. SMA Type IV is the mildest form and has its onset in adulthood. Progressive muscle weakness is the main feature but generally does not affect life expectancy [146].

### 6.2. Genetic Diagnosis and Reproductive Risk

The vast majority of SMA cases are caused by biallelic loss of function variants in the *SMN1* gene, located on chromosome 5q13.2 (‘5q SMA’). The rare non-5q forms account for less than 4% of cases and are not covered in this discussion. The disease mechanism involves the deficient expression of the Survival Motor Neuron (SMN) protein, which is essential for the maintenance and survival of motor neurons [147]. A closely related pseudogene, *SMN2*, which presents high sequence homology to *SMN1* (>99%), also resides in the 5q12.2-q13.3 region. Although *SMN2* is almost identical to *SMN1*, it produces a much lower amount of functional protein due to alternative splicing that frequently excludes exon 7 [147]. The number of copies of *SMN2* varies among individuals and influences the severity of SMA; more copies of *SMN2* are generally associated with milder forms of the disease [148]. Consequently, the variability in SMA (type I–IV) is largely determined by the number of copies of *SMN2* [145,147]. Deletions are the most common *SMN1* pathogenic variants (92% of cases). In a small percentage of cases (about 4%), a pathogenic SNV is found, usually in compound heterozygosity with a deletion [145].

Genetic testing is crucial to confirm the diagnosis by identifying pathogenic variants in the *SMN1* gene; evaluation of *SMN2* CNVs provides further information on genotype–phenotype correlation. *SMN1* variants (and *SMN2* CNVs) can be identified by gene deletion–duplication analysis such as RT-PCR and MLPA. In a few cases where only a heterozygous deletion of *SMN1* is detected, sequencing analysis is required to identify SNVs. SMA is inherited in an autosomal recessive manner, and biallelic variants in *SMN1* are necessary to manifest the disease. Hence, the reproductive risk for a couple of carriers is 25% per pregnancy. However, some factors complicate the interpretation of genetic analysis, counseling, and risk estimation. Notably, around 2% of affected individuals have a de novo variant, meaning one parent will not be a carrier [149]. In these cases, the recurrence risk is considered low; however, the possibility of germline mosaicism in one parent cannot be ruled out. Furthermore, in carrier screening, it is important to consider the possibility of false negative results. The test correctly identifies individuals with two copies of *SMN1*. Still, it cannot discriminate between the in trans (one copy on each allele) or in cis (two copies on the same allele) configuration. A “2+0” carrier (also known as silent carrier), with two *SMN1* copies on one chromosome and none on the other (in cis configuration), may yield a false negative result. Hence, “2+0” carriers present a comparable recurrence risk as the classic “1+0” carriers but are not detected by routine testing. Indeed, the detection rate of carrier screening drops significantly in populations with a high frequency of “2+0” carriers, such as sub-Saharan Africans (70% vs. approximately 95% in other populations) [145]. Carrier tests generally do not include the assessment of point variants. Therefore, even with a negative result (two copies of *SMN1* detected), the possibility of being a carrier of point variants or having a 2+0 genotype remains [145]. For individuals from pan-ethnic populations, it is worth noting that even after a negative screening test, a residual risk of about 1 in 500 remains [150]. For affected individuals, the recurrence risk is generally considered low. An affected individual will pass one of the two mutated alleles to their offspring, making the child a healthy carrier. However, the unaffected partner has an approximate pre-test probability of 1 in 50 of being a carrier (equal to the general population carrier frequency in the absence of consanguinity or high inbreeding between partners). Consequently, the recurrence risk for these couples is approximately 1 in 200. After a negative carrier screening result in the unaffected partner, the post-test probability of having an affected child remains higher than the general population (estimated as 1 in 2000 compared to 1 in 10,000). In the context of preconception and prenatal counseling, it is nowadays essential to discuss the available reproductive options and current genetic therapies for SMA. Prospective parents should understand the advancements in SMA treatments, which can significantly impact disease management and quality of life, and hence their choice about family planning and reproductive options.

### 6.3. Current Treatment and Emerging Therapies

Currently, three targeted therapies have been approved for the treatment of SMA in the USA and EU, fundamentally altering the natural history of the disease [151,152,153,154,155]. These therapies include Nusinersen (Spinraza^®^), Onasemnogene abeparvovec (Zolgensma^®^), and Risdiplam (Evrysdi^®^) [153]. Nusinersen is an antisense oligonucleotide, which modifies the splicing of the *SMN2* gene to increase the production of full-length SMN protein. It is delivered by lumbar puncture every four months following an initial loading phase. Onasemnogene abeparvovec is a gene therapy delivered intravenously as a one-time dose that introduces a functional copy of the *SMN1* gene into motor neuron cells through an AAV. It is currently approved for pediatric patients under the age of two years. Risdiplam is an orally administered small molecule splicing modifier that increases SMN protein production by promoting exon 7 inclusion in *SMN2* transcripts. This therapy is approved for a wider range of patients, from infants to adults.

These therapies have significantly improved patient outcomes, leading to extended survival, improved motor function, and the achievement of developmental milestones previously unattainable for many individuals with SMA. However, they are not curative, and a disease burden remains, particularly in patients treated later in the disease course. Residual motor deficits, respiratory difficulties, and mobility limitations continue to affect the quality of life of treated individuals. Furthermore, access to these therapies is not universal. Age restrictions (as seen with Onasemnogene abeparvovec), challenges with intrathecal administration (as with Nusinersen), and differences in healthcare resources can limit treatment availability. Furthermore, the high costs associated with these therapies present an additional barrier to widespread access [153].

### 6.4. Prenatal Diagnosis and Preimplantation Genetic Testing

For PND, the same methodologies applied in postnatal settings can be used. Couples may be offered invasive diagnosis through CVS or AC to find the variants in *SMN1*, or alternatively PGT within an assisted reproductive technology program. The test provides an effective method to offer couples the chance to have a pregnancy with a child unaffected by SMA by reliably distinguishing carrier embryos from non-carrier embryos. PGT can be applied to most couples (with deletions or point variants), and segregation of the mutant allele of the *SMN1* gene can be performed both with a direct method (variant analysis) and an indirect method (risk haplotype analysis) [156].

### 6.5. Fertility and Management of Pregnancy

While some neuromuscular pathologies may negatively affect fertility, there is no consistent data on SMA patients with reduced fertility [103]. With the advancement of therapies, more women with SMA are reaching childbearing age and considering the possibility of having children. When planning a pregnancy, a multidisciplinary approach involving all preparatory steps is recommended for the couple [7]. Preconception counseling is crucial for patients with a clinical and genetic diagnosis of SMA to receive multidisciplinary counseling and discuss reproductive risks and implications of pregnancy, disease progression, and available support options. Before pregnancy, it is important to assess the patient’s respiratory function, as muscle weakness can compromise lung capacity. Cardiological evaluation is also necessary, as muscle weakness can impact the cardiovascular system. Management during pregnancy should involve a multidisciplinary team, including neurologists, obstetricians, pulmonologists, cardiologists, nutritionists, and physiotherapists [157]. In affected women, the incidence of fetal complications such as polyhydramnios, IUGR, or macrosomia is not increased compared to the reference population [158]. Routine visits and continuous monitoring of SMA progression and fetal development are essential for proper pregnancy management. Since respiratory impairment is one of the main concerns during pregnancy, monitoring respiratory function is critical. Forced vital capacity and oxygen saturation should be monitored regularly. The use of non-invasive ventilation may be necessary in some cases. Nutritional management is also crucial, as SMA patients may have difficulty swallowing and maintaining adequate nutritional status. A balanced diet and, if necessary, enteral feeding may be used. Management of muscle weakness is essential during pregnancy too. Physiotherapy can help maintain muscle function and improve quality of life. There may be a worsening of muscular symptoms during pregnancy, so the use of assistive devices, such as wheelchairs, may be necessary for mobility. Psychological support is important to address the anxiety and stress associated with pregnancy and the management of SMA. The mode of delivery should be planned by considering the patient’s respiratory and muscular function; vaginal delivery would not have contraindications, although a cesarean section would be recommended due to muscle weakness and possible respiratory compromise [159]. The risk of premature birth is increased. The choice of anesthesia should be carefully considered, with epidural or spinal anesthesia potentially preferred over general anesthesia, which carries greater respiratory risks [6]. After delivery, continuous monitoring of the mother is essential to manage any respiratory or nutritional complications. Physiotherapy and respiratory assistance may be necessary to support postpartum rehabilitation.

In conclusion, the management of a patient with SMA requires a coordinated approach addressing both the medical needs of the mother and the developmental needs of the fetus, considering that around 40% of pregnant women experience an exacerbation of weakness during pregnancy [158]. A multidisciplinary team and a personalized care plan are essential to optimize the outcomes for both. Close collaboration among various specialists and careful planning can significantly improve the quality of life of both the mother and child. A standardized multidisciplinary approach is essential to effectively address every possible scenario, including the management of anesthesiologic risks, respiratory function, and chronic pain. It is also important to regularly evaluate and monitor motor and respiratory function before, during, and after pregnancy [157].

## 7. Limb–Girdle Muscular Dystrophy (LGMD)

### 7.1. Epidemiology and Clinical Features

The term Limb–Girdle Muscular Dystrophy (LGMD) encompasses a heterogeneous group of inherited muscular dystrophies characterized by progressive weakness and wasting of the muscles, primarily affecting the pelvic and shoulder girdles. Overall, LGMD is a rare disorder, whose prevalence varies significantly depending on the studied population, with estimates ranging from 1 in 14,500 to 1 in 123,000 individuals [160,161]. The age of onset is usually adolescence to early adulthood but is highly variable, ranging from early childhood to late adult life, depending mainly on genetic subtypes. Early symptoms often include impaired walking, unusual gait, and difficulties with running. As the disease progresses, patients may require assistance walking and may eventually become wheelchair-dependent [162,163]. The progression of LGMD varies according to the subtype and can lead to significant disability. Approximately 60.8% of patients with LGMD experience loss of ambulation [164]. For early childhood-onset LGMD, 71.1% of patients become non-ambulatory, typically by a mean age of 17.7 years [164]. Ventilatory involvement is variable, with some forms, such as LGMD2I, having a high risk for respiratory failure, while others, such as LGMD2B/Dysferlinopathy, rarely affect respiratory muscles [163]. Cardiac involvement also varies, being common in some forms like LGMD2I but rare in others such as LGMD2A/Calpainopathy and LGMD2B/Dysferlinopathy [163].

### 7.2. Genetic Diagnosis and Reproductive Risk

LGMD is associated with different causal genes involved in dystrophin–glycoprotein complex, sarcomeric proteins, glycosylation, and vesicle trafficking and nuclear functions [160]. LGMD2A (Calpainopathy), caused by pathogenic variants in the *CAPN3* gene, presents with variable severity, from mild to severe, with early involvement of the pelvic muscles followed by the shoulder muscles [160,163]. LGMD2B (Dysferlinopathy), caused by variants in the *DYSF* gene, is marked by early weakness and atrophy of the pelvic and shoulder muscles, generally without respiratory or cardiac involvement [160,163]. Sarcoglycanopathies (LGMD2C-2F) involve variants in genes encoding sarcoglycan proteins, commonly present with muscle weakness in the lower and upper girdles and variable severity, and are often associated with cardiomyopathy [160,163]. Sarcoglycanopathies are notably prominent among early childhood onset forms (68% of severe childhood forms) [165]. Based on the mode of inheritance, LGMD is classified as LGMD1 when it follows an autosomal dominant inheritance pattern and as LGMD2 when it follows an autosomal recessive inheritance pattern. Autosomal recessive forms are the most prevalent, accounting for approximately 90% of all LGMD cases [160]. The most common subtypes include LGMD2A (Calpainopathy), LGMD2B (Dysferlinopathy), and LGMD2C-2F (Sarcoglycanopathies). Autosomal dominant forms are relatively rare (less than 10% of cases worldwide); the most common dominant subtypes include LGMD1A (Myotilinopathy), LGMD1B (Laminopathy) and LGMD1C (Caveolinopathy) [160]. Considering the rarity of each individual genetic cause, the reproductive risk for individuals affected by autosomal recessive forms is only marginally increased with respect to the general population, assuming that the partner is not a known carrier of a pathogenic variant in the same gene. An affected individual will pass one of the two mutated alleles to their offspring, who will be a carrier of the condition but not affected, as the presence of biallelic variants is necessary to manifest the disease. However, special considerations should be made in cases of consanguinity or among populations with a high carrier frequency. For dominant forms, the recurrence risk for each conception is 50%, although it is not possible to precisely predict the clinical phenotype due to incomplete penetrance and variable expressivity. The available diagnostic strategies involve a comprehensive approach that includes clinical evaluation, muscle biopsy, and genetic analysis [166]. For genetic testing and molecular confirmation, NGS is generally applied to sequence multiple LGMD-associated genes simultaneously, providing high-resolution data for detecting point variants, insertions, deletions, and splice site variants [160,163]. Additionally, MLPA or QF-PCR can be utilized to detect CNVs in LGMD-related genes, which are common, for example, in the *SGCA* and *SGCG* genes. Furthermore, for variants that may affect splicing, RNA analysis can be performed by RT-PCR to confirm the presence of specific variants at the transcript level.

### 7.3. Prenatal Diagnosis and Preimplantation Genetic Testing

PND and PGT are generally feasible once the pathogenic variant(s) have been identified in the family, in the carrier partner, or in the affected partner. PND involves targeted testing for the known variants(s), with the method used varying based on the type of pathogenic variant identified (e.g., Sanger sequencing, RT-PCR, MLPA, etc.). PGT is typically less frequently applied in cases where one partner is affected by LGMD, as most LGMD cases follow a recessive inheritance pattern, generally not posing an increased recurrence risk. However, in couples coming from regions with a high carrier frequency or where consanguinity with the partner is established, it is advisable to test the healthy partner. As with other monogenic disorders, once it is established that both members of the couple are carriers of at least one causative variant, PGT can be used. For both dominant and recessive forms, direct analysis of the specific variant(s) and concurrent indirect confirmation analysis by identifying the at-risk haplotype through flanking marker assessment are generally advisable to reduce the risk of false negatives due to amplification failure or allele dropout. For indirect analysis in PGT, a preliminary setup phase is necessary to confirm technical feasibility. This stage requires the availability of other family members to identify informative genetic markers, accurately reconstruct the at-risk haplotype, and reduce the risk of misdiagnosis.

### 7.4. Fertility and Pregnancy

Data on obstetric complications in LGMD are limited, with most of the information coming from small case series and individual case reports. From the available data, there appears to be an increased risk of prolonged labor and operative delivery [143,167]. Additionally, there may be an increased risk of abnormal fetal presentation. Awater et al. (2012) reported breech presentation in 26.7% of cases, a higher risk not consistently observed in other studies [6]. Furthermore, increased rates of cesarean sections have been observed [7,149], although this was not confirmed by the recent study by Libell et al. [150]. Overall, there is documented worsening of symptoms during pregnancy in approximately 50% of cases [6,143,167], and these symptoms typically do not resolve after delivery. Specifically, Moore et al. observed a deterioration in balance in 91% of LGMD2B patients, with 57% experiencing falls during pregnancy [2]. Furthermore, approximately 40% of the patients were unaware of their LGMD diagnosis before pregnancy, complicating management [143,167]. Case reports, such as von Guionneau et al. (2019), highlight the worsening of cardiac function during pregnancy in LGMD patients, particularly those with LGMD2I, which require specialized management including regular cardiac evaluations and multidisciplinary care with cardiologists and obstetricians [168]. In conclusion, data are limited, and comparing them across studies is challenging due to differences in study designs, LGMD subtypes analyzed, and the limited number of cases described, making it difficult to draw generalized conclusions. Nonetheless, it is crucial for reproductive medicine professionals, particularly obstetricians, to be aware of the potential complications in patients with LGMD. These include prolonged labor, the need for operative delivery and cesarean section, an increased risk of gestational hypertension, and deterioration of cardiac function and muscular symptoms.

## 8. Amyotrophic Lateral Sclerosis (ALS)

### 8.1. Epidemiology and Clinical Features

Amyotrophic Lateral Sclerosis (ALS) is a neurodegenerative disorder marked by progressive paralysis of muscles resulting from the degeneration of motor neurons in the primary motor cortex, the corticospinal tracts, the brainstem, and the spinal cord. The degenerative process leads to progressive paralysis, affecting functions such as speaking, swallowing, and breathing [169]. ALS affects approximately 2–3 people per 100,000 annually, with a prevalence of about 5–7 per 100,000, representing the most common adult-onset motor neuron disease. While it can occur at any age, it is more common between the ages of 40 and 70 years, with a slight male predominance (male-to-female ratio of 1.5:1) [170].

The disease presents with phenotypic heterogeneity, with about two-thirds of patients with typical ALS presenting with the spinal form of the disease (whose manifestations begin in the limbs) and symptoms associated with focal muscle weakness and atrophy, starting distally or proximally in both the upper and lower limbs. Spasticity progresses gradually, causing weakening and atrophy of the limbs, affecting manual dexterity and gait [171]. The remaining one third of patients develop bulbar onset of ALS, which typically manifests with dysarthria and dysphagia for solid foods or liquids. Limb symptoms usually develop almost simultaneously with bulbar symptoms and, in most cases, appear within the first two years. The paralysis is progressive and leads to death from respiratory failure on average about 2–3 years after onset in bulbar-onset cases and 3–5 years after onset in limb-onset ALS cases [172]. Although ALS affects primarily the motor system, it can also involve broader frontotemporal degeneration, which may lead to varying degrees of cognitive and behavioral dysfunction [173]. Various family studies have shown a significant pathophysiological and clinical overlap between ALS and frontotemporal dementia (FTD), especially in cases related to variants in the *C9ORF72*, *TARDBP*, *FUS*, and *VCP* genes [174,175,176,177].

### 8.2. Genetic Diagnosis and Reproductive Risk

ALS is a multifactorial disease with a complex genetic architecture that includes monogenic, oligogenic, and polygenic contributions. While some cases are caused by single-gene variants, many involve multiple genetic variants and environmental factors that interact over time [171]. It is estimated that about 10% of ALS cases are familial, and in at least half of these, a monogenic cause can be identified. To date, more than 30 genes have been associated with ALS [171]. The most common hereditary form is associated with hexanucleotide repeat (GGGGCC) expansion located between the noncoding exons 1a and 1b of the *C9ORF72* gene, also involved in the pathogenesis of frontotemporal dementia, with a pathogenic threshold of 61 repeats [178]. Less frequently, pathogenic variants are found in the *SOD1* gene (21q22.11), encoding superoxide dismutase-1, in *TARDBP* (1p36.22), which encodes TAR DNA-binding protein 43 (TDP-43), in *FUS* (16p11.2), encoding an RNA-binding protein, and in *VCP* (9p13.3), which encodes a valosin-containing protein [179]. The pathophysiological mechanism of ALS is not fully understood. Multiple interconnected mechanisms are involved, including impaired RNA metabolism, disrupted protein homeostasis (proteostasis), defects in nucleocytoplasmic transport, mitochondrial dysfunction, oxidative stress, and inflammation. The most common monogenic forms of ALS are primarily due to gain-of-function mechanisms [180].

The diagnosis of ALS is primarily clinical, based on the patient’s history and neurological examination. To standardize diagnostic criteria, the most commonly used are the revised Escorial criteria. Additional assessments include EMG to assess muscle function, magnetic resonance imaging to exclude other pathologies, and evoked potentials [181]. Genetic testing can identify specific variants, providing a definitive diagnosis and allowing genetic counseling and recurrence risk assessment. Target analysis for repeat expansion in *C9ORF72* should be performed first, as it represents the most common monogenic form. If the *C9ORF72* test is negative, the next step involves multigene panel sequence analysis using NGS. Additional analyses may be considered, such as exome or genome sequencing, CMA, or repeat expansion testing for other genes associated with ALS (i.e., *ATXN2*, *ATXN1*) [182].

The most common forms of monogenic ALS are inherited in an autosomal dominant manner, including the *C9ORF72*, *SOD1*, *TARDBP*, *FUS*, and *VCP* variants. In these cases, affected individuals have a 50% chance of transmitting the causative variant to their offspring. The clinical presentation can vary widely, even among family members with the same variant, due to variable expressivity and incomplete penetrance. However, in a high proportion of cases, even with a positive family history, it is not possible to identify a monogenic cause. In such cases, it is not possible to provide useful prognostic information for clinical management or an accurate estimation of recurrence risk or reproductive options to prevent transmission.

### 8.3. Prenatal Diagnosis and Preimplantation Genetic Testing

Once a molecular diagnosis is established in the affected partner (or a family member), PND or PGT can be considered. For prenatal diagnosis, the same methodologies applied in postnatal settings can be used on the DNA obtained from CVS or AC. PGT can be applied to most couples by direct or indirect analysis [156]. Indirect analysis is performed using polymorphic markers, such as STRs or SNPs. Typically, an indirect approach is applied alongside direct analysis to increase the accuracy of testing, enabling detection of allele dropout, contamination, and recombination. This combined approach is particularly useful when the partner is at risk of a hereditary form of ALS but prefers not to undergo genetic testing (non-disclosure test). As seen with FSHD, methods used for repeat expansion analysis (e.g., Southern blot analysis) are unsuitable for PGT due to the limited amount of DNA available. In cases involving repeat expansions, such as *C9ORF72*, indirect analysis alone is applied. However, indirect analysis alone is not suitable for de novo cases, as it requires affected family members to identify the at-risk haplotype accurately during setup.

### 8.4. Fertility and Pregnancy

Since ALS rarely affects individuals of reproductive age, there is limited data available about the impact of the disease on fertility, the risk of obstetric complications, the progression of the disease during pregnancy, the actual incidence of the disease, and the frequency of monogenic forms in this age group. Based on available reports, it is likely that fertility is not affected. Lunetta et al. (2014) highlight in their cohort that 16.3% of affected women developed ALS during reproductive age (19–49 years) and 2% within the last three months of pregnancy or one month after delivery (“ALS in pregnancy”). Furthermore, in cases of ALS during pregnancy, pathogenic variants in the *SOD1* gene were significantly more common (2/5 patients) [183]. Few studies have examined the reciprocal relationship between ALS progression and pregnancy. Some case reports suggest rapid or severe progression in patients during and after pregnancy [167,168]. In their recent review, Hamad et al. collected reports of ALS overlapping pregnancy dating back to 1980, identifying 38 cases [184]. This review shows that, although 95% of pregnancies result in the birth of a live baby, pregnancy in these patients is often associated with rapid and severe disease progression [167]. However, this association could be due to ascertainment bias, whereby cases with particularly rapid or severe courses are more likely to be reported. Nonetheless, a link cannot be excluded, considering that pregnancy, through hormonal changes, neuro-inflammation, increased metabolic demand, and vascular and muscular strain, could trigger the onset of the disease and accelerate its progression [183,184]. In contrast, a retrospective study conducted by Yang et al. on a total of 52 reproductive-aged patients between 19 and 49 years, 49 of whom had at least one pregnancy, suggests that pregnancy is not associated with disease progression, which did not differ significantly from that of the male control group. Furthermore, the study highlighted a higher frequency of ALS-associated gene variants in patients diagnosed within one year before or after pregnancy, compared to those diagnosed more than a year after pregnancy. Notably, *SETX* variants were more frequent [185]. However, information on the genetic background of these patients remains limited. In many of the reported cases, genetic analyses performed on patients are often not specified. Among the cases collected by Hamad et al., genetic testing information was provided for only 10 out of 36 patients; of these, tests were carried out in 9 patients, revealing a pathogenic or likely pathogenic variant in 5 cases [183,184,186,187,188]. All these cases were documented after 1993, the year the first gene associated with ALS was identified [189]. Currently, the evidence available is limited. However, genetic testing should be considered for all patients who develop ALS during reproductive age. Regarding the clinical management of pregnancy, the key areas to monitor include: (i) ventilatory function, monitoring of forced vital capacity and oxygen saturation is crucial, with non-invasive ventilation provided if necessary; (ii) nutritional support, due to swallowing difficulties and weight loss (a tailored diet, nutritional supplements, or enteral nutrition may be required); (iii) management of muscle weakness, (physiotherapy and occupational therapy can help maintain functionality and improve quality of life); and (iv) psychological support, to help manage anxiety and depression. Regarding the mode of delivery, since ALS does not affect smooth muscle function, including uterine muscles, there is no absolute contraindication to vaginal delivery for these patients. However, given the increased respiratory effort required during the expulsive phase of vaginal delivery, a cesarean section is considered a safer option in cases of ventilatory failure [184,190]. Similarly, the assessment of respiratory function is crucial when managing anesthesia. Regional anesthesia, such as spinal or epidural anesthesia, is generally preferred over general anesthesia to reduce the risk of respiratory complications [6,190]. Due to the increased risk of preterm delivery [184], careful fetal monitoring is essential. This includes regular ultrasound examinations to monitor fetal growth and detect any signs of preterm labor. If preterm delivery, which is also common [184], is likely, corticosteroids may be administered.

In conclusion, there is no solid evidence on pregnancy in patients with ALS. There appears to be an increased risk of preterm delivery and cesarean section [183,184]. In addition, a more rapid progression of the disease is possible in patients with monogenic forms [184]. A multidisciplinary approach involving neurologists, obstetricians, pulmonologists, cardiologists, nutritionists, physiotherapists, and neonatologists is crucial to optimizing outcomes for both the mother and the fetus [191].

## 9. Discussion

The increasing availability, both in general diagnostics and specifically in prenatal and preimplantation diagnosis, of advanced and accessible molecular DNA sequencing techniques has greatly expanded the pool of individuals with monogenic diseases or carriers of these conditions, who are able to turn to medically assisted reproductive centers with the intent of preventing transmission of their at-risk condition to their offspring. This is particularly relevant in the context of NMDs, which are associated with a significant impairment in quality of life. Individuals with NMDs are generally able to make informed reproductive decisions and are therefore potentially holders of a strong interest in exploring available reproductive options. In addition, the development and commercial introduction of highly effective therapies for monogenic conditions previously associated with early lethality, such as SMA type I and *DMD* [68,69,151,152,153,154,155], encourage the prediction that in the future an even greater proportion of these patients will be able to reach reproductive age and consequently require access to assisted reproductive techniques.

Therefore, it is essential that healthcare professionals are well-prepared to manage the available assisted reproduction techniques in order to provide at-risk couples with all the necessary information to make the most informed and conscious decision, support them through the process of ART when needed, and effectively manage the pregnancy and peripartum period. In this context, the field of NMDs poses several challenges, both general and specific. Knowledge of the underlying genetic alterations and the available molecular techniques is essential for the accurate diagnosis and referral to the appropriate assisted reproduction treatment [2,3]. In particular, patients must be made aware of the limitations of preimplantation diagnosis techniques with respect to the genetic disorder under investigation [3,4]. For example, in conditions such as FSHD2 or in cases involving uninformative *DMPK* alleles, the PGT outcomes may be unclear, and it is essential to discuss alternative reproductive options, such as prenatal diagnosis and adoption [94,120]. Further complexity arises from an ethical standpoint in couples at increased risk of transmitting disorders such as ALS and certain subtypes of CMT and LGMD, in which the late onset makes the natural history of the disease less predictable, complicating reproductive choices [4].

Once pregnancy is achieved, a journey begins for women with hereditary NMDs in which the health issues normally associated with pregnancy can be exacerbated by comorbidities specific to these conditions [2,5,6,7]. Placental abnormalities, symptomatic urinary tract infections, worsening of cardiac and pulmonary function, and even exacerbation or onset of myopathy or other symptoms related to the underlying pathology are eventualities to be considered in NMD patients, especially those wheelchair-bound [2,5,6,7]. Abnormal fetal presentation, preterm labor, and risk of postpartum hemorrhages are also more frequent [4,5,6]. Although literature on obstetrical issues related to hereditary NMDs is relatively limited, larger case series from phenotypically similar conditions, such as spinal cord injury or other chronic physical disabilities, may offer helpful guidance [192,193]. The management of pregnancy should be carefully monitored and tailored based on the patient’s condition. Considering the need for specific expertise depending on the type of disability, a multidisciplinary approach is strongly recommended. Vaginal birth should not be discouraged in all circumstances but should be carried out only in an advanced tertiary care center.

## 10. Conclusions

NMDs present significant challenges in the context of reproductive medicine. These hereditary disorders, though individually rare, collectively have a high overall prevalence [1] and can critically impact the quality of life, particularly concerning reproductive health and pregnancy management. The clinical complexities of NMDs during pregnancy necessitate a multidisciplinary approach that includes genetic counseling, preconception planning, and careful monitoring throughout pregnancy and the postpartum period. Diagnosing these conditions accurately is paramount to informing recurrence risks, reproductive options, and appropriate management strategies to mitigate obstetric complications and potential neonatal risks [3,4]. Overall, managing pregnancies in women with NMDs demands an in-depth understanding of the molecular mechanisms, clinical manifestations, and potential complications associated with these conditions. Genetic testing, including PGT and PND, offers valuable tools for prospective parents but must be approached with a clear understanding of each condition’s specific challenges and limitations [2,3]. This review underscores the importance of tailored care and the integration of genetic and reproductive counseling to optimize outcomes for both parents and their offspring.

## Figures and Tables

**Table 1:** Summarized genetic, reproductive, and pregnancy-related aspects of NMDs discussed in this review. CMT = Charcot–Marie–Tooth disease; *DMD* = Duchenne Muscular Dystrophy and dystrophinopathies; DM = Myotonic Dystrophy; FSHD = Facioscapulohumeral Muscular Dystrophy; SMA = Spinal Muscular Atrophy; LGMD = Limb Girdle Muscle Dystrophy; ALS = Amyotrophic Lateral Sclerosis; MOI = Mode of Inheritance; AD = Autosomal Dominant; XL = X-linked; AR = Autosomal Recessive; OdP = Onset or diagnosis during or soon after pregnancy frequently reported; Y = yes; N = no; RR = Recurrence Risk; PGT = Preimplantation Genetic Testing; PND = Prenatal Diagnosis; SGA = Small for Gestational age referring to the neonate at birth; het = heterozygous; hemi = hemizygous; homo = homozygous; P = pathogenic; * variable expressivity and reduce penetrance; † = possible anticipation and more severe phenotype (congenital DM); § = assuming the partner is healthy and not carrier of pathogenic variant and/or permissive allele; ° = females typically present with a milder phenotype and could be asymptomatic; + = increased risk; - = risk not increased; +/− = increased risk for some studies, not increased for others.

	CMT		*DMD*	DM		FSHD		SMA	LGMD		ALS
	AD	XL		DM1	DM2	FSHD1	FSHD2		LGMD1	LGMD2	
**Gene defect**	*PMP22* dup or het P variant in *PMP22*, *MFN2* or *MPZ*	Hemi or het P variant in *GJB1*	Hemi or het P variant in *DMD*	(CTG)n repeat expansion (>50) in *DMPK*	(CCTG)n repeat expansion (>75) in *CNBP*	D4Z4 repeat contraction (1–10) + 4qA permissive allele	*SMCHD1*, *DNMT3B* or *LRIF1* het P variant + 4qA permissive allele	*SMN1* biallelic P variants	Het P variant in causative genes	Biallelic P variant in *CAPN3*, *DYSF* or other causative genes	(GGGGCC)n repeat expansion (>60) in C9ORF72 or Het P variant in *SOD1*, *TARDBP*, *VCP,* or *FUS*
**MOI**	AD	XL	XL	AD	AD	AD	digenic	AR	AD	AR	AD
**Genetic testing**	*PMP22* deletion/duplication analysis ± sequence analysis	*PMP22* deletion/duplication analysis ± sequence analysis	*DMD* deletion/duplication analysis (i.e., MLPA)	Targeted analysis for CTG repeats expansion in *DMPK*	Targeted analysis for CCTG repeats expansion in *CNBP*	Southern blot (D4Z4 allele size) ± haplotype analysis	D4Z4 methylation analysis + sequence analysis	*SMN1* deletion/duplication	Multigene panel sequence analysis	Multigene panel sequence analysis	Multigene panel sequence analysis + targeted analysis for repeat expansion in *C9ORF72*
**Fertility**	Not affected	Not affected	Not affected	Affected	Affected (male only)	Not affected	Not affected	Not affected	Not affected	Not affected	Not affected
**RR**	50% *	50% °	50% °	50% †	50% *	50% *	25% §	Negligible §	50% *	Negligible §	50% *
**PGT**	Technically feasible	Technically feasible	Technically feasible	Technically feasible (indirect analysis only)	Technically feasible (indirect analysis only)	Technically feasible (indirect analysis only)	Not available	Technically feasible	Technically feasible	Technically feasible	Technically feasible
**PND**	Technically feasible	Technically feasible	Technically feasible	Technically feasible	Technically feasible	Technically feasible	NOT available	Technically feasible	Technically feasible	Technically feasible	Technically feasible
**Ectopic** **pregnancies**	-	-	-	+	-	-	-	-	-	-	-
**Miscarriages**	-	-	-	-	-	-	-	-	-	-	-
**Hypertensive disease**	-	-	-	-	-	-	-	-	+	+	-
**Polyhydramnios**	-	-	-	+	-	-	-	-	-	-	-
**Placenta abnormalities**	+/−	+/−	-	+	-	-	-	-	-	-	-
**Symptomatic urinary tract** **infections**	+/−	+/−	-	+	+	-	-	-	-	-	-
**OdP**	n	n	n	y	y	y	y	n	y	y	n
**Lung function worsening**	-	-	-	+	-	-	-	+/−	-	-	+
**Cardiac function worsening**	-	-	+	-	-	-	-	+/−	+	+	-
**Exacerbation of myopathy**	+	+	+/−	+	-	+	+	+	+	+	+
**Impaired nutritional status**	-	-	-	-	-	-	-	+/−	-	-	+
**Abnormal fetal presentation**	+/−	+/−	-	+	-	-	-	-	+/−	+/−	-
**Preterm labor**	+/−	+/−	-	+	+	-	-	+	-	-	+/−
**Instrumental** **delivery**	+/−	+/−	-	+	-	+	+	+	+	+	-
**Cesarean delivery**	+/−	+/−	-	+	-	+	+	+	+	+	+/−
**SGA**	-	-	-	+	+	+/−	+/−	-	-	-	-
**Critical neonate and perinatal death**	-	-	-	+ (congenital form)	-	-	-	-	-	-	-
**Peri-partum Hemorrhage**	+/−	+/−	-	+	-	-	-	-	-	-	-

## Data Availability

No new data were created or analyzed in this study.
